# Integrated Transcriptome Analysis Reveals Plant Hormones Jasmonic Acid and Salicylic Acid Coordinate Growth and Defense Responses upon Fungal Infection in Poplar

**DOI:** 10.3390/biom9010012

**Published:** 2019-01-02

**Authors:** Jie Luo, Wenxiu Xia, Pei Cao, Zheng’ang Xiao, Yan Zhang, Meifeng Liu, Chang Zhan, Nian Wang

**Affiliations:** 1 College of Horticulture and Forestry Sciences, Huazhong Agricultural University, Wuhan 430070, China; luojie@mail.hzau.edu.cn (J.L.); xwxrencongzhong@163.com (W.X.); caoly991@163.com (P.C.); xiaoza0815@163.com (Z.X.); mint_19@163.com (Y.Z.); mf_swag@163.com (M.L.); aurora0045@163.com (C.Z.); 2 Hubei Engineering Technology Research Center for Forestry Information, Huazhong Agricultural University, Wuhan 430070, China

**Keywords:** poplar, defense regulation, integrated transcriptome analysis, co-expression network construction, jasmonic acid, salicylic acid

## Abstract

Plants have evolved a sophisticated system to respond to various stresses. Fungal attack or infection is one of the most important biotic stresses for most plants. During the defense response to fungal infection, the plant hormones jasmonic acid (JA) and salicylic acid (SA) play critical roles. Here, gene expression data on JA/SA treatments and *Melampsora larici-populina* (MLP) infection were generated. Integrated transcriptome analyses of these data were performed, and 943 genes in total were identified as common responsive genes (CRG). Gene ontology (GO) term analysis revealed that the genes from CRG are generally involved in the processes of stress responses, metabolism, and growth and development. The further cluster analysis of the CRG identified a set of core genes that are involved in the JA/SA-mediated response to fungal defense with distinct gene expression profiles upon JA/SA treatment, which highlighted the different effects of these two hormones on plant fungal defenses. The modifications of several pathways relative to metabolism, biotic stress, and plant hormone signal pathways suggest the possible roles of JA/SA on the regulation of growth and defense responses. Co-expression modules (CMs) were also constructed using the poplar expression data on JA, SA, *M.* *larici-populina*, *Septoria musiva*, and *Marssonina brunnea* treatment or infection. A total of 23 CMs were constructed, and different CMs clearly exhibited distinct biological functions, which conformably regulated the concerted processes in response to fungal defense. Furthermore, the GO term analysis of different CMs confirmed the roles of JA and SA in regulating growth and defense responses, and their expression profiles suggested that the growth ability was reduced when poplar deployed defense responses. Several transcription factors (TFs) among the CRG in the co-expression network were proposed as hub genes in regulating these processes. According to this study, our data finely uncovered the possible roles of JA/SA in regulating the balance between growth and defense responses by integrating multiple hormone signaling pathways. We were also able to provide more knowledge on how the plant hormones JA/SA are involved in the regulation of the balance between growth and plant defense.

## 1. Introduction

Plants have evolved a sophisticated system to respond to various abiotic and biotic stresses. Fungal attack or infection is one of the most important biotic stresses for most plants. When encountering fungal attack or infection, plants typically produce a number of small chemical compounds to activate their self-protection system. Recently, a number of studies have reported that jasmonic acid (JA) and salicylic acid (SA) are two important small chemical compounds that play important roles in activating the plant self-protection system [1,2]. Both of these small chemical compounds are also plant hormones, which regulate plant growth [3,4,5]. These two plant hormones typically play different roles against fungal attack or infection. Jasmonic acid is induced by the lipoxygenase pathway when plants are challenged by stress [6,7,8]. A number of genes are subsequently activated by JA signals through two distinct branches [2]. The first branch is the MYC branch, in which MYC-related transcription factors and JA-responsive genes are activated. The second branch is the ERF branch, in which apetala2/ethylene response factor (AP2/ERF) transcription factors are the key regulators. Once one of the two branches is activated, downstream defensive or resistance genes are downregulated or upregulated by genes in the branch pathways. Unlike JA, SA is induced upon the perception of microbe-associated molecular patterns, and is required for hypersensitivity and immune responses in plants [9,10]. The critical role of SA as an immune signal has also been well studied. In general, pathogens with a biotrophic lifestyle are more sensitive to SA-induced defenses, whereas necrotrophic pathogens and herbivorous insects are combatted through JA-mediated defenses [2]. Moreover, pathways regulated by JA and SA also interact when plants encounter biotic stresses [11,12,13]. Although there have been many studies characterizing the mechanisms underlying the JA/SA regulation of plant defense, detailed biological processes that are activated by JA/SA remain unknown.

Given the development of high-throughput sequencing technology, increasingly large genomic data are publicly available. Of these genomic data, short reads produced from RNA-Seq and gene intensity data produced in microarray assays are the two most important for gene expression. Given that large amounts of these data were generated for similar plants and treatments, it is therefore possible to integrate all of these data together and unearth more information for further studies. In soybean, an integrated transcriptome analysis was performed to reveal the utility of different types of transcriptomic data for comparison [14]. Of the methods used for integrating transcriptomes, co-expression network construction is one of the most popular strategies that is used for analysis. The basic idea of co-expression networks is that genes that participate in similar biological processes could have similar expression profiles in a given set of expression samples [15]. In plants, a number of studies have been conducted to dissect the complexity of gene–trait regulation. For example, in *Arabidopsis*, the co-expression of genes in six accessions identified 53 genes that are responsible for aluminum tolerance [16]; the co-expression of seed maturation also identified regulators linking seed storability to biotic defense-related pathways [17]. Therefore, with increasing amounts of available genomic data, integrated transcriptome analysis, especially for co-expression network construction, is very powerful for plant genomic studies.

Poplar (*Populus* spp.) is one of the most important tree species. In 2006, the genome of *Populus. trichocarpa* was released [18]. Given the characteristics of this tree, including its small genome size, high-quality genome sequence, easy propensity for producing transgenic plants, and its use as a common source of wood, poplar is considered a model plant for tree species. Fungal diseases, including leaf rust, spots, and stem canker, highly reduce the value of poplar. Recently, a number of studies have been performed to uncover how poplar trees deploy their defense system in response to fungal attack. Due to its high-quality genome, transcriptome analysis would be one of the fastest methods to identify the genes that are involved in the poplar defense system. Two typical studies reported hundreds of genes that were responsive to *Melampsora* defense regulation in poplar using a *Populus* 15.5 K complementary DNA microarray [19,20]. RNA-Seq data were also generated for poplar infected by *Melampsora larici-populina*, *Septoria musiva*, and *Marssonina brunnea* [21,22,23]. All of these data were generated in different individual experiments, and no comparison was performed.

In this study, we generated a large set of gene expression data for poplar responses to JA/SA/*M. larici*-*populina* (MLP) treatments. Using an integrated transcriptome analysis of all these data, a number of mechanisms underlying poplar defense regulation was uncovered. A large portion of JA/SA-responsive genes also exhibited responses to fungal infection in poplar, which revealed that JA/SA play important roles in the regulation of plant defense. Moreover, a set of common responsive genes (CRG) were identified, and were enriched in the key pathways that are important for plant growth, metabolism, stress responses, and plant hormone signaling transduction. Co-expression analysis of a number of transcriptomic datasets from public databases confirmed the results that are obtained in the current study, collectively demonstrating the roles of JA/SA on regulating the balance between growth and defense responses by integrating multiple hormone signaling transduction processes. Therefore, according to the results of this study, we were able to provide more knowledge on how the plant hormones JA/SA are involved in the balance between growth and plant defense regulation.

## 2. Materials and Methods

### 2.1. Plant Materials and Phytohormone Treatments

The Chinese poplar hybrid variety “NL895” (*P.* × *euramericana*) was used as plant material in this study. The “NL895” variety was produced from the hybridization between *Populus. deltoides* cv. “Lux” (I-69) and “I-45” (*P.* × *euramericana*). Three-leaf tissue culture plants of “NL895” that exhibited similar growth were grown in three-liter pots containing a sand:peat (50:50, *v*/*v*) mixture. These “NL895” plants were grown in a greenhouse with a 16/eight-hour photoperiod, and the light intensity was 12,000 Lx. The temperatures of the greenhouse were set at 28 °C and 25 °C for day and night, respectively. Plants were watered twice weekly and fertilized with Hoagland’s solution every other week.

To apply phytohormone treatments to poplar leaves, a procedure was performed that was similar to that reported in our previous study [24]. Briefly, 10-leaf plants that exhibited similar growth were selected as the plant materials, and leaves with LPIs (leaf plastochron indices) ranging from four to six on each plant were treated with methyl jasmonate (MeJA) or SA solution. The solutions were prepared to final concentrations of 0.2 mM and 0.5 mM for MeJA and SA, respectively. Leaves were sprayed on both sides until the leaf surfaces began to form small drops. Leaf samples were collected at 0 h, 2 h, 6 h, 12 h, and 24 h after treatment, and were immediately stored at −80 °C. Treated leaves from each individual plant were pooled and considered one biological sample, and three replicates were performed for each treatment. To avoid plant day/night rhythms affecting gene transcription, we initiated all treatments at 8:00 to allow collecting all of the samples during the daytime in the greenhouse (the lights were activated at 6:00 and deactivated at 22:00). Samples collected at 2 h, 6 h, 12 h, and 24 h after MeJA treatment were referred to as JA2, JA6, JA12, and JA24, respectively, and samples collected at two hours, six hours, 12 h, and 24 h after SA treatment were referred to as SA2, SA6, SA12, and SA24, respectively. Samples collected from untreated plants and at the treatment start were referred to as controls.

### 2.2. RNA Extraction and Transcriptome Sequencing

Total RNA was isolated using an RNeasy Plant Mini Kit (DP432, TIANGEN Biotech (Beijing) Co., Ltd., Beijing, China) according to the manufacturer’s protocol. The quality of RNA was first tested by agarose gel electrophoresis to ensure that clear bands were visible. The RNA integrity number (RIN) of all of the samples was also tested using an Agilent Bioanalyzer 2100 (Agilent Technologies, Santa Clara, CA, USA). Samples with sufficient quality were sent for RNA-Seq library construction and submitted to an Illumina HiSeq 2500 (Illumina, Inc., San Diego, CA, USA) sequencing platform. All of the raw data were deposited in the National Center for Biotechnology Information (NCBI) Sequence Read Archive (http://www.ncbi.nlm.nih.gov/sra/) under bioproject accession PRJNA511770. Copies of the above RNA samples were also stored at −80 °C for further gene expression analysis.

### 2.3. Quantitative Real-time PCR Assay

To confirm the RNA-Seq results, 10 genes were selected to examine the consistency of their expression profiles after MeJA and SA treatments using RNA-Seq and Quantitative Real-time PCR (qRT-PCR) assays. The gene information and corresponding primers are listed in Table S1. A One-Step genomic DNA Removal and cDNA Synthesis SuperMix kit (Trans, Beijing, China) were used for cDNA synthesis. To confirm the correct amplification of primer pairs, the cDNA PCR products of the primers listed in Table S1 were also sequenced before qRT-PCR assays. The LightCycler 96 (Roche, Basel, Switzerland) platform and the FastStart Essential DNA Green Master mix (Roche) were used for all of the qRT-PCR assays. All of the PCRs were performed using the two-step qRT-PCR procedure, and three replications per sample were performed to reduce the experimental error. Sample quantification cycle (Cq) values were determined and standardized relative to the two reference genes (*ACTIN* and *UBQL*, Table S1). The 2^−ΔΔCq^ method was used to calculate the relative gene expression based on the qRT-PCR data [25].

### 2.4. Bioinformatic Analysis

In the integrated transcriptome analysis, the information regarding *Melampsora*-responsive genes was downloaded from the original installment [19,20]. All of the IDs of the responsive genes were transferred from V1 to V3 by the correspondence between poplar genome annotation version one and three. The raw RNA-Seq data were quality-trimmed and quality-filtered using Trimmomatic software with default parameter settings [26]. Clean reads for each sample were mapped onto poplar genome annotation version 3 by Tophat2 software with default parameter settings [27]. To identify JA-responsive and SA-responsive genes, a two-step analysis procedure was conducted. First, values of fragments per kilobase per million mapped reads (FPKM) were calculated using Cufflinks software [27]. Second, the differentially expressed genes were identified using EdgeR software with a false discovery rate (FDR) that was less than 0.05 under the ‘glm’ model [28]. To avoid low-expression genes in both treatments and in the control, only genes with FPKM values greater than five in at least one sample were retained for further analyses. 

The poplar genes were annotated based on their homologous genes from *Arabidopsis*, which were provided by the Phytozome database (version 12.0) [29]. The gene ontology (GO) term and KEGG analyses were conducted in R with packages named “clusterProfiler” and “pathview” [30,31]. The significant GO terms of CRG in biological process were further reduced with REVIGO (http://revigo.irb.hr/) [32] and visualized in Cytoscape (version 3.5.0) [33] The subcellular location analysis of CRG was performed within the SUBA4 (http://suba.live/) [34]. The heatmap was drawn in R with the package “pheatmap” [35]. The gene expression levels of different clusters were generated with the vioplot package in R [36]. Gene classification and the pathway visualization of metabolism and biotic stress were conducted using Mapman software (version 3.0.0) according to the user manual (version 3.0.0) [37]. Bubble diagrams representing the enriched GO terms were generated in R with the package “ggplot2” [38].

For co-expression network construction, four datasets were used in this analysis. The first dataset was obtained from our above RNA-Seq experiment and included 18 individual samples; the corresponding bioproject accession number is PRJNA511770. The second dataset was obtained from our previous study in which four samples were used for poplar lncRNA identification during *Melampsora* infection of poplar leaves. RNA samples isolated from leaves treated with the *Melampsora* uredospore suspension or diluted water after 48 h. The corresponding bioproject accession number is PRJNA354475 [23]. The third dataset was obtained from a study on the comparative expression analysis of resistant and susceptible *Populus* clones inoculated with *S. musiva*. Briefly, leaves of the two resistant (DN34, *P. deltoides* × *P. nigra*; NM6, *P. nigra* × *maximowiczii*) and two susceptible clones (DN164, *P. deltoides* × *nigra*; NC11505, *P. maximowiczii* × *trichocarpa*) were inoculated with *S. musiva,* and then RNA was isolated from leaf samples harvested after 48 h of inoculation. This study included 16 samples, and the original data are deposited in the Sequence Read Archive under bioproject accession number PRJNA229492 [21]. The fourth dataset was obtained from the analysis of the transcriptomes of *M. brunnea* and infected poplar [22]. Conidia of *M. brunnea* were sprayed onto the abaxial surface of the poplar “NL895” leaves, and RNA was isolated from leaf samples harvested after 48 h of inoculation. This study released data on nine individual samples; the corresponding bioproject accession number is PRJNA248151. All of the above original RNA-Seq reads were quality-trimmed and quality-filtered using Trimmomatic software with default parameter settings [26]. Clean reads were used for gene FPKM calculation using RESM software [39]. Genes with FPKM values greater than five in at least one sample were used for co-expression network construction. The FPKM of genes with no FPKM data in a given sample was set to 0. The WGCNA (weighted gene correlation network analysis) R package was then used to perform the co-expression network construction [15]. A soft threshold was determined by which >80% of the model fit to scale-free topology and low mean connectivity. The gene number in each module was set from 30 to 5000. The co-expression network of individual co-expression modules (CMs) was visualized using Cytoscape (version 3.5.0) [33].

## 3. Results and Discussion

### 3.1. Identification of Jasmonic Acid-Responsive and Salicylic Acid-Responsive Genes

The JA and SA phytohormones are involved in plant biotic defense regulation. Treating plants with JA, SA, or their derivatives, MeJA and methyl salicylic acid (MeSA), respectively, could modulate the gene expression profiles regarding pathogenic or herbivore damage to plants. Therefore, we performed a transcriptome assay by treating leaves of the poplar hybrid “NL895” with MeJA and SA. Gene expression profiles upon treatment with MeJA and SA were generated from RNA-Seq data. To improve the understanding of this work, JA2, JA6, JA12, and JA24 represented samples treated with MeJA after 2 h, 6 h, 12 h, and 24 h, respectively, and SA2, SA6, SA12, and SA24 represented samples treated with SA after 2 h, 6 h, 12 h, and 24 h, respectively.

Compared with control samples, a number of genes exhibited different regulation upon MeJA or SA treatment within 24 h. For example, the number of differentially expressed genes (fold change greater than two for at least one time point and q-value < 0.05) was 3930 after MeJA treatment, whereas 6226 genes were differentially expressed genes after SA treatment (Table S2). Thus, the differentially expressed genes upon the time series under MeJA and SA treatments were defined as JA-responsive and SA-responsive genes, respectively.

To confirm the gene expression of JA-responsive and SA-responsive genes, qRT-PCR assays were performed to test the gene expression profiles upon MeJA and SA treatments for nine genes. The relative fold-changes between each treatment and the control samples were calculated for both RNA-Seq and qRT-PCR data. The consistency between these two types of expression data was revealed based on correlation coefficient number, with an *r*^2^ value equal to 0.85 and a *p*-value of less than 0.0001 (Figure S1). Clearly, this high *r*^2^ value suggested that our RNA-Seq data were very reliable for further analyses.

Gene ontology term analysis of JA-responsive and SA-responsive genes revealed that these two exogenous stimuli were enriched in a large number of common pathways, which were involved in responses to diverse environmental stimuli, hormone-related metabolism processes, and regulation processes (Table S3). Thus, it’s highly possible that these two phytohormones may share common pathways to regulate biotic and abiotic stresses, including fungal defense.

### 3.2. Characterization of the Roles of Jasmonic Acid-Responsive and Salicylic Acid-Responsive Genes in the Regulation of Melampsora Defense by Comparative Transcriptome Analysis

**Figure 1:**
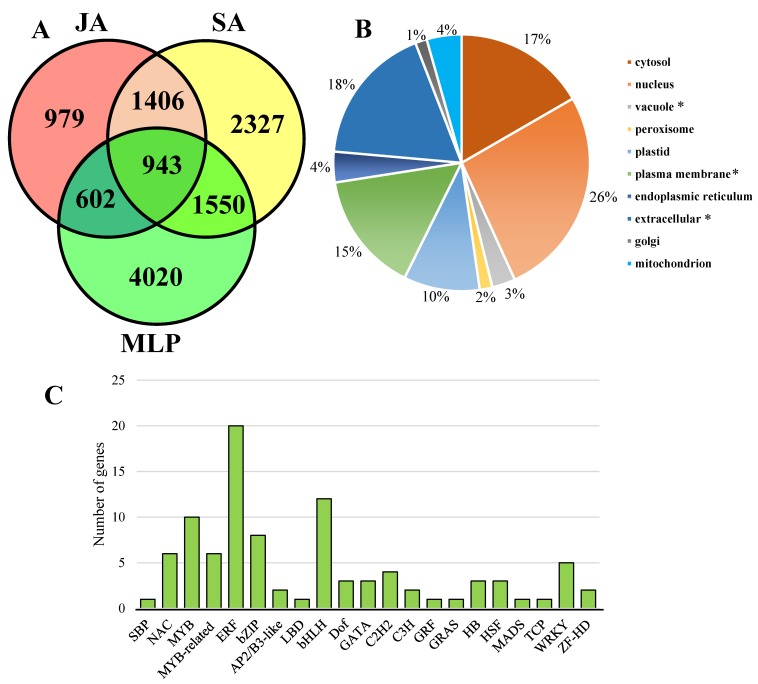
Venn diagram represented the number of specific and overlapping genes identified as jasmonic acid (JA)-responsive, salicylic acid (SA)-responsive, and *Melampsora larici-populina* (MLP)-responsive genes (**A**). The proportion of cellular locations of proteins encoded by common responsive genes (CRG, (**B**)). The star behind the cellular location indicates that the *p*-value of the hypergeometric distribution test was less than 0.05. The gene numbers in each transcription factor family were showed in CRG(**C**).

To test the possible roles of JA-responsive and SA-responsive genes in the regulation of *Melampsora* defense, we compared JA-responsive and SA-responsive genes with *Melampsora*-responsive genes. After 48 h of infection with *M. larici-populina*, gene-reprogramming events resulted in 2676 and 4439 upregulated and downregulated genes, respectively (Table S4). Then, these genes were defined as MLP-responsive genes. To identify JA/SA-responsive genes that participated in the fungal defense, Venn analysis was performed to obtain the list of genes that commonly exist in the JA-responsive, SA-responsive, and MLP-responsive genes (Figure 1A). Among the 7115 MLP-responsive genes, 943 genes exhibited a response to JA and SA treatments, and were designated as CRG. In addition, 979 genes were exclusively responsive to JA treatment; meanwhile, 2327 genes were exclusively responsive to SA treatment (Figure 1A, Table S5). Thus, the CRG may represent the core machinery of the JA/SA-responsive genes that are involved in fugal defense, and the list of CRG was used for further analysis.

Interestingly, the cellular location analysis of CRG revealed that proteins encoded by CRG largely accumulated in the nucleus (about 26%), implying that the list of CRG may include a large number of transcription factors (Figure 1B). To verify this hypothesis, transcription factors were screened in the CRG according to PlantTFDB [40], which allowed us to identify 95 (ca. 10% of CRG) transcription factors in total (Figure 1C). These transcription factors belonged to 21 gene families, and ERF, MYB/MYB-related genes, and bHLH include the highest number of represented genes compared with other gene families (Figure 1C, Table S6). These results highlight the roles that these transcription factor gene families (e.g., ERF, MYB/MYB-related genes, and bHLH) play in the phytohormone-mediated fugal defense.

Indeed, according to previous studies, these several types of transcription factors (TFs) play important roles in plant disease resistance. For example, ERF is involved in JA-mediated defense responses [41,42]. A large number of ERFs has been identified as regulators of disease resistance in many plant species, such as *AtERF2*, *AtERF4 AtERF6*, *AtERF14*, and *ORA59* in *Arabidopsis* [43,44,45,46,47]; *TaERF3* and *TaPIEP1* in wheat [48,49]; *GmERF3* in soybean [50]; and *OsERF3*, *OsERF922*, and *OsEREBP1* in rice [51,52,53]. Regarding bHLH, the subgroup bHLH IIId transcription factors (bHLH3, bHLH13, bHLH14, and bHLH17) in *Arabidopsis* were found to act as targets of JAZ proteins and negatively regulate JA-mediated plant defense and development [54]. *PtrWRKY23*, *PtrWRKY73*, and *PtWRKY89* are involved in disease resistance or defense regulation when overexpressed in transgenic poplar or *Arabidopsis* [55,56,57]. These results suggest the potential effectiveness for identifying candidate genes for given biological processes. Moreover, we also found that the majority of the 95 TFs were novel genes in poplar, and these genes have not been cited in previous reports or have not been functionally characterized. For example, the three WRKY genes, *Potri.017G088300*, *Potri.011G070100,* and *Potri.018G008500*, were not *PtrWRKY23*, *PtrWRKY73*, and *PtWRKY89*. Therefore, these results suggest that the TFs that were identified in this study could be considered candidate genes in poplar disease resistance. The functional characterization of these TFs is a necessary next step in poplar disease-resistant studies.

**Figure 2:**
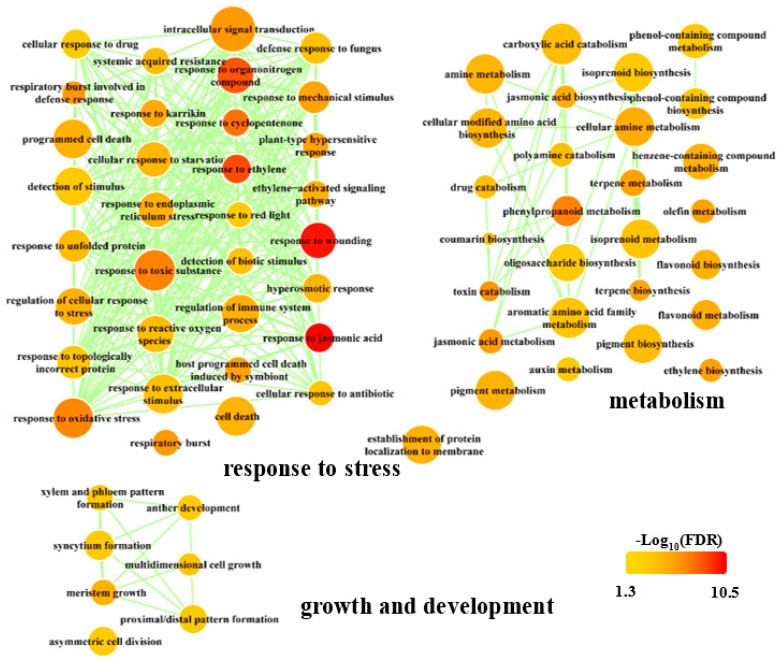
Significantly enriched gene ontology (GO) terms in biological process. The results were obtained in R using the “clusterProfiler” package, reduced with REVIGO (http://revigo.irb.hr/) and visualized in Cytoscape. The color indicated the significance of GO terms, and node size represented the frequency of the GO term. Only significant GO terms were shown. FDR, false discovery rate.

To further understand the function of CRG, GO functional analysis was performed. Regarding the 943 CRG, 144 biological GO processes were enriched with an “FDR” < 0.05 (Table S7). These 144 biological processes were reassigned to 68 terms using REVIGO software based on their relationships (Figure 2). Interestingly, these terms can be roughly divided into three subgroups, i.e., response to stress, metabolism, and growth and development. In the group of responses to stress, many key terms relative to phytohormone and fugal stimuli were significantly enriched, such as “regulation of immune system process”, “defense response to fungus”, “response to ethylene”, “host programmed cell death induced by symbiont”, “response to jasmonic acid”, “response to extracellular stimulus”, “respiratory burst involved in defense response”, “systemic acquired resistance”, and “response to wounding” (Figure 2, Table S7). The systemic acquired resistance in plants involved contributions induced by JA and SA upon pathogenic attacks [58]. In the category of metabolism, many pathways involved in hormone and secondary metabolism were significantly enriched (Figure 2, Table S7). Regarding growth and development, the terms relative to cell division and tissue formation were enriched (Figure 2, Table S7). Interestingly, several genes encoding essential auxin signaling components, such as SAUR, GH3, and PIN proteins, were found in the CRG (Table S5). In line with this, the processes that were enriched in the category of growth and development were consistent with the roles of auxin in plant growth and development [59,60]. This may represent the crosstalk between auxin and JA/SA that regulates plant growth and development upon biotic and abiotic stresses.

Regarding cellular components in GO terms, “plant-type cell wall”, “anchored component of membrane”, and “apoplast” were enriched (Table S7). These results were consistent with the cellular location analysis, indicating that proteins encoded by CRG were enriched in the extracellular compartment (Figure 1B). Forty-eight hours after infection is the key time point for poplar deploying defense regulation against *Melampsora* in poplar [19,20]; thus, the recruitment of cell wall-related components and proteins may help plants defend against the fungal infection of the cell.

The GO terms analysis clearly demonstrated that the CRG identified in this study might function as coordinators in balancing the processes of stress response and growth and development upon fungal infection. 

### 3.3. Jasmonic Acid and Salicylic Acid May Exhibit Different Effects on Plant Fungal Defense

**Figure 3:**
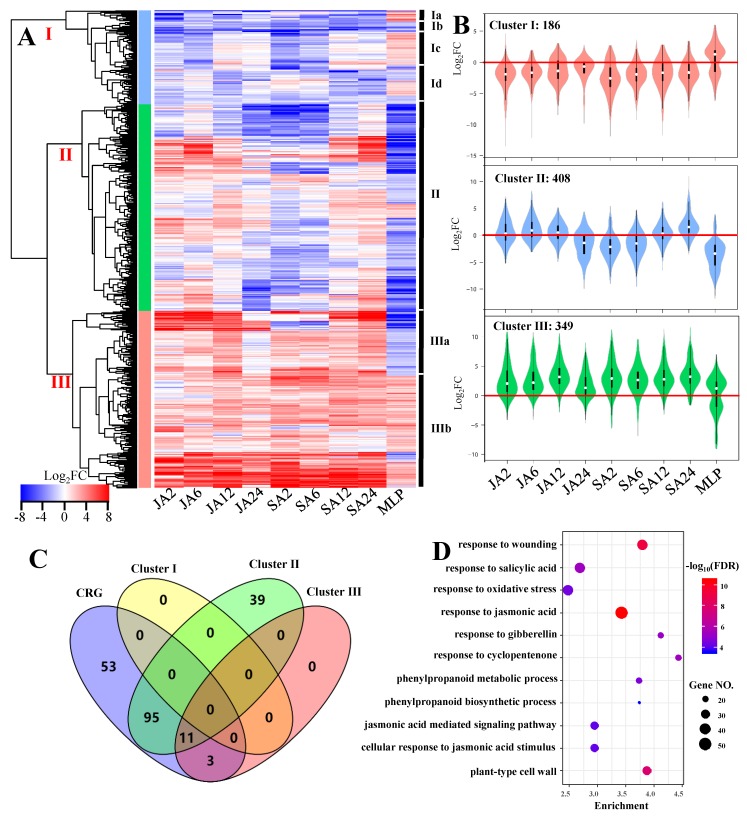
Heatmap clustered the CRG into three clusters based on their gene expression profiles (**A**). The average expression levels of genes from different clusters under different treatments (**B**). Venn diagram presents the overlapping GO term detected from CRG, Cluster I, Cluster II, and Cluster III (**C**). The significantly enriched GO terms commonly detected in CRG, Cluster II, and Cluster III (**D**). FDR, false discovery rate; FC, folds change.

As mentioned above, we identified a group of JA/SA-responsive genes that may be important for plant resistance of fungal infection. As reported previously, JA and SA initiate different signaling pathways and have distinct roles in fungal defense processes. However, interactions between these two phytohormones also contribute to the plant fungal defense [61]. Here, we identified 943 genes that are potentially involved in fungal defense; however, their expression profiles were distinctly different among different treatments (Figure 3A). As shown in Figure 3A, the gene expression levels of MLP were generally inhibited, and were distinct from those of JA/SA treatments. These CRG can be divided into three major clusters based on their expression levels upon treatments (Figure 3A). Cluster I included 186 genes, which were globally downregulated under both JA and SA treatment along the time series, and slightly upregulated by MLP (Figure 3A,B, Table S5). According to their gene expression profiles in MLP, genes clustered into Cluster I can be further divided into four subgroups, i.e., Cluster Ia–d (Figure 3A). In Cluster II, 408 genes displayed different reprograming profiles during the experimental time course under JA and SA treatments (Figure 3A,B, Table S5). More specifically, these genes were stimulated and inhibited by JA and SA, respectively, during the experimental time course (Figure 3A,B). However, these genes were generally inhibited by MLP (Figure 3A,B). In Cluster III, gene expression levels of JA/SA exhibited the same trends and were globally stimulated (Figure 3A,B). This cluster can be further separated into two distinct subgroups based on the gene expression levels of MLP, and genes from Cluster IIIb were consistently upregulated under all of the detected conditions (Figure 3A).

Gene ontology term analysis further confirmed that genes from Cluster II were more related to the roles that CRG played in plant fungal defense, as they shared 106 terms with CRG (Figure 3C, Table S7). Thus, the genes from Cluster II represent the core machinery of JA/SA in response to plant fungal defense. No significant GO terms were detected in the genes from Cluster I; meanwhile, genes in this cluster exhibited similar behaviors as JA and SA in the time series (Figure 3A–C, Table S7). However, some significant GO terms were found in its subgroups, for instance, two GO terms that were relative to water response were enriched in Cluster Ia, and several genes that participated in plant hormone signal transduction were identified in Cluster Id by KEGG analysis (Table S7). Cluster III shared 11 significant GO terms with Cluster II and CRG, and these shared common GO terms that were related to the core machinery of hormone-mediated plant fungal defense (Figure 3C,D, Table S7). Moreover, the genes from Cluster IIIb were more likely relative to defense response than those from Cluster IIIa, as revealed by GO enrichment analysis (Table S7). Thus, considering the uniform gene reprograming profiles and significant enriched pathway uncovered by GO term analysis, the genes from Cluster IIIb may represent the common machinery of JA and SA in plant fungal defense.

In conclusion, the identification of CRG suggest that JA and SA may mount similar pathways regulating plant fungal defense; thus, the different gene expression profiles upon time-series JA and SA treatments in the core list may explain the distinct effects of JA and SA on plant fungal defense. This conclusion was consistent with the previous results obtained from *Arabidopsis*, which revealed the antagonistic roles played by JA and SA in regulating plant immune signaling through the same regulatory pathway [11]. On the other hand, attention should be given to the genes from Cluster IIIb, as they exhibited similar response profiles among different times and treatments. The consistent behaviors highlighted the notion that these genes potentially participate in defense processes, which are important for plants to gain systemic acquired resistance. The GO term analysis further confirmed this notion given that all of the significant GO terms are correlated with the roles of hormones in biotic and abiotic stresses.

### 3.4. Common Responsive Genes are Involved in Key Pathways that are Important for Plant Fitness

**Figure 4:**
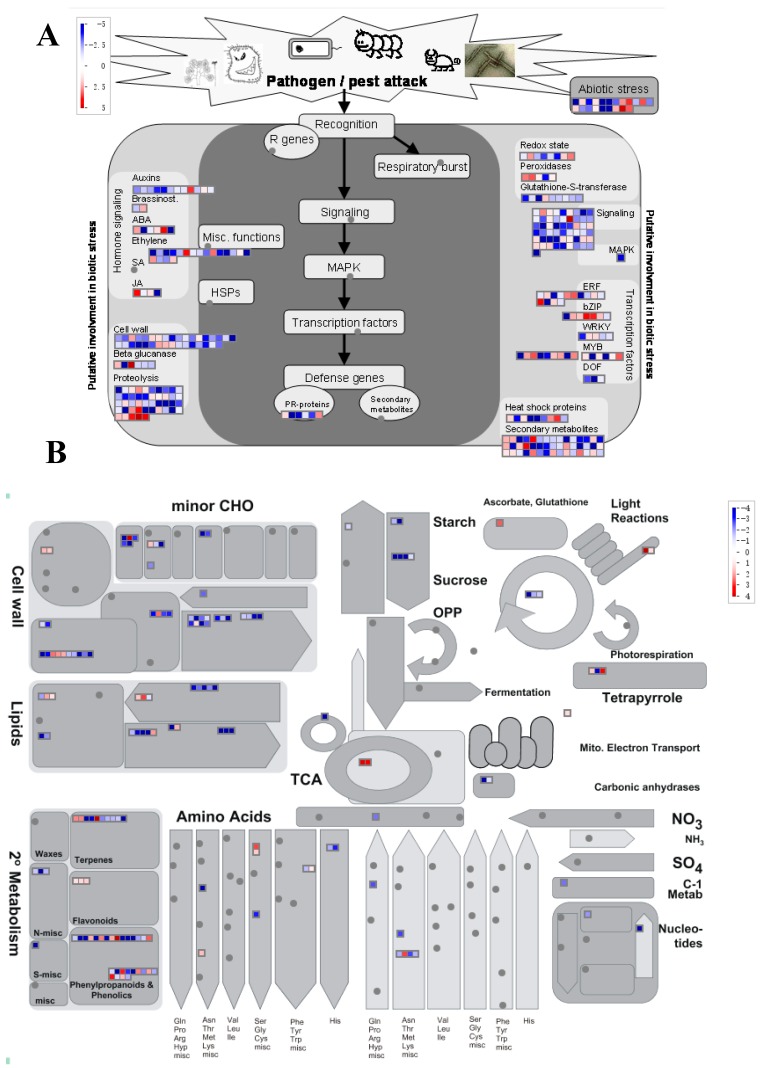
Common responsive genes assigned to biotic stress (**A**) and metabolism (**B**) categories based on Mapman software. The red to blue color indicates the fold changes of *Melampsora larici-populina* (MLP)-responsive genes. The biotic stress pathway consisted of the genes that participated in abiotic stress, redox, signaling, transcription factors, heat shock proteins, Pathogenesis protein, hormone relative genes, as well as the genes implicated in cell wall and proteolysis. For metabolism pathway, genes involved in carbon fixation and metabolism, cell wall, lipids metabolism, major nutrition uptake, and metabolism, as well as second metabolism, were included.

**Figure 5:**
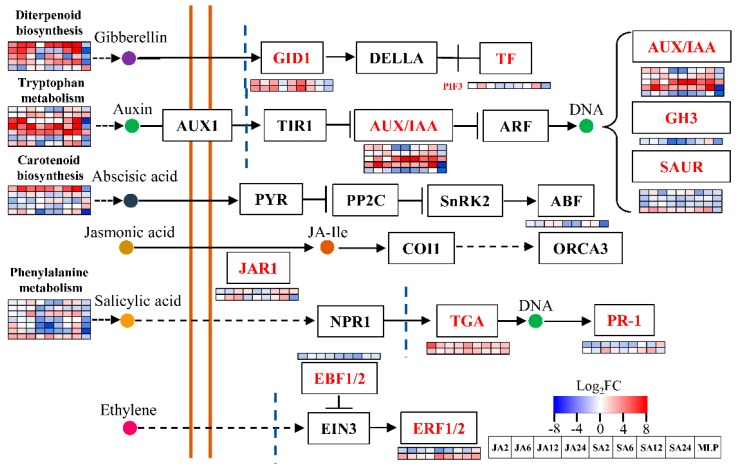
Common responsive genes assigned to plant hormone signal transduction and their upstream metabolism pathways. This figure was adapted from the KEGG pathway of plant hormone signal transduction. The gene expression levels of each treatment were shown in different columns as indicated in figure; the different rows represent the different genes. The colors indicate the gene expression levels.

As revealed by GO term analysis, the processes enriched in the CRG generally belonged to stress responses, metabolism, and growth and development. Thus, using Mapman software, genes involved in metabolism and biotic stress were assigned (Figure 4). A total of 331 (ca. 35%) genes played roles in biotic stress (Figure 4A). A large number of CRG was assigned to subgroups of signaling, transcription factor, secondary metabolites, proteolysis, the cell wall, and hormone signaling (Figure 4A, Table S5). In total, 154 genes were assigned to metabolism categories using Mapman (Figure 4B). Consist with this result, KEGG analysis revealed that CRG are enriched in several metabolism pathways, e.g., “starch and sucrose metabolism”, “phenylpropanoid biosynthesis”, “diterpenoid biosynthesis”, and “carotenoid biosynthesis” (Table S7). Coincidently, these pathways or relative pathways were involved in phytohormone metabolism. For example, “diterpenoid biosynthesis” and “carotenoid biosynthesis” represent the metabolism pathways for gibberellin and abscisic acid (Figure 5). In addition, “phenylpropanoid biosynthesis” is a pathway that is closely related to “phenylalanine metabolism” and is responsible for SA synthesis (Figure 5). Thus, the changes in exogenous phytohormones are eventually transformed into gene programming events that integrate multiple hormone signals to regulate plant responses to fungal infection. For instance, the changes in several genes encoding essential components of auxin signaling, such as AUX/IAA, GH3, and SAUR [60], potentially modified the growth and development processes mediated by auxin during the duration of the plant fungal defense. Consistent with this hypothesis, the GO term analysis identified a set of auxin-related growth and development processes (Table S7). Furthermore, JA interacts with GA to balance plant growth and defense processes through their common JAZ and DELLA regulatory proteins [62,63]. JAR1 (GH3.11) in CRG is an auxin-induced gene that participates in pathogen defense. JAR1 converts JA into the biologically active jasmonoyl -isoleucine (JA-Ile) form, which affects the functions of JAZ. In addition, several genes involved in the GA signaling pathway were also changed, for example, two genes encoding GID1 and one encoding PIF3 (Figure 5, Table S5). Taken together, our data suggest that the modifications of the metabolism and signal transduction of the key phytohormones may regulate the balance between plant growth and the defense capacities against pathogen infection by integrating multiple phytohormone signals.

### 3.5. Co-Expression Networks Confirmed the Possible Roles of Jasmonic Acid and Salicylic Acid in Response to Fungal Defense by Balancing Growth and Biotic Stress Responses

**Figure 6:**
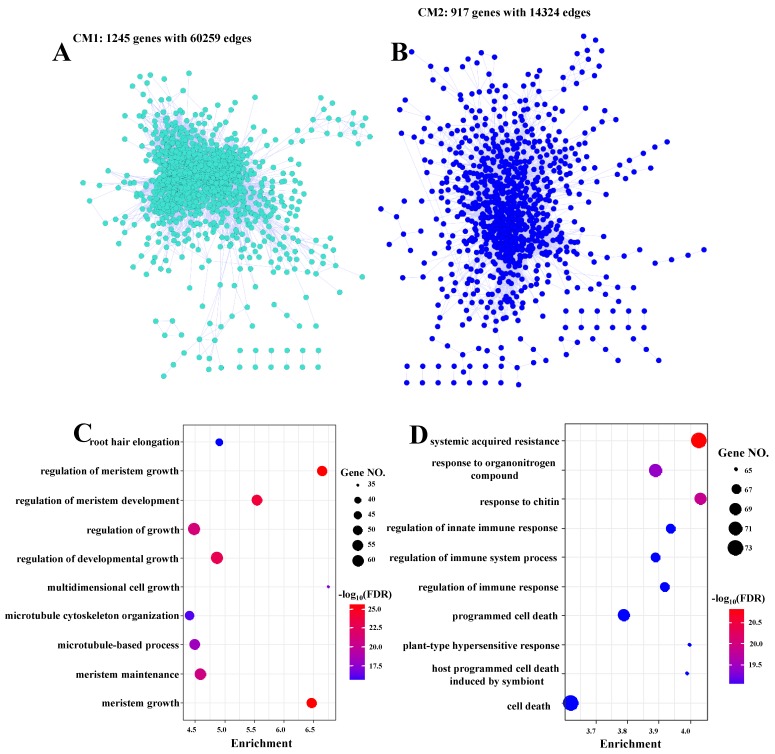
Gene co-expression networks of co-expression module 1 (CM1, (**A**)) and co-expression module 2 (CM2, (**B**)). The nodes and edges represented the genes and the significant correlations between genes, respectively. The top 10 significantly enriched GO terms in biological process in CM1 (**C**) and CM2 (**D**).

Recently, co-expression network construction has become a powerful integrated transcriptome tool to analyze a large microarray of RNA-Seq datasets [64,65]. A meta-based transcriptomic approach was performed to construct a co-expression network using 47 individual samples collected from RNA-Seq public data, and all of the samples were produced for fungal infection or JA/SA treatments (see Materials and Methods (M&M)). Therefore, we performed a co-expression network construction to investigate the roles of JA-responsive and SA-responsive genes in the regulation of fungal defense. Using the 11827 JA/SA/MLP-responsive genes from the above RNA-Seq experiments as edge genes, we calculated their expression levels for each sample collected from four datasets (See M&M). Weighted gene correlation network analysis was employed to perform the co-expression network construction [15]. In total, 23 CMs were constructed, and 7333 genes were arranged into these 23 modules (Figure S2A, Table S8). The gene numbers in the 23 CMs ranged from 37 to 1561, and co-expression module 1 (CM1) and co-expression module 23 (CM23) contained the highest and lowest gene numbers (Table S8), respectively. In the 23 CMs, JA/SA/MLP-responsive genes and CRG were majorly distributed in CM1 and co-expression module 2 (CM2), and JA-responsive genes were enriched in CM5 as expected (Figure S2B). Thus, CM1 and CM2 represented the major influence of JA, SA, and MLP on reprogramming gene expression profiles. The co-expression networks of CM1 (removing the genes without edges) and CM2 contain 1245 and 917 genes with 60259 and 14324 edges, respectively (Figure 6A,B). The GO term analysis clearly demonstrated the roles of CM1 and CM2 in growth and stress responses, respectively (Figure 6C,D, and Table S9). In addition, GO terms relative to metabolism were also significantly enriched in both CM1 and CM2 (Table S9). Interestingly, GO terms related to transport, such as “nitrate transport”, “proline transport”, “ammonium transport”, and “amino acid import”, were enriched in CM2 (Table S9). Consistent with this finding, the GO terms of CRG were also enriched in several nitrogen starvation-related terms (Table S7). Collectively, these data clearly suggested a role of nitrogen metabolism in fungal defense [66]. In summary, the co-expression network analysis clearly confirmed the roles of JA/SA in fungal defense via regulating the balance between plant growth and defense response.

**Figure 7:**
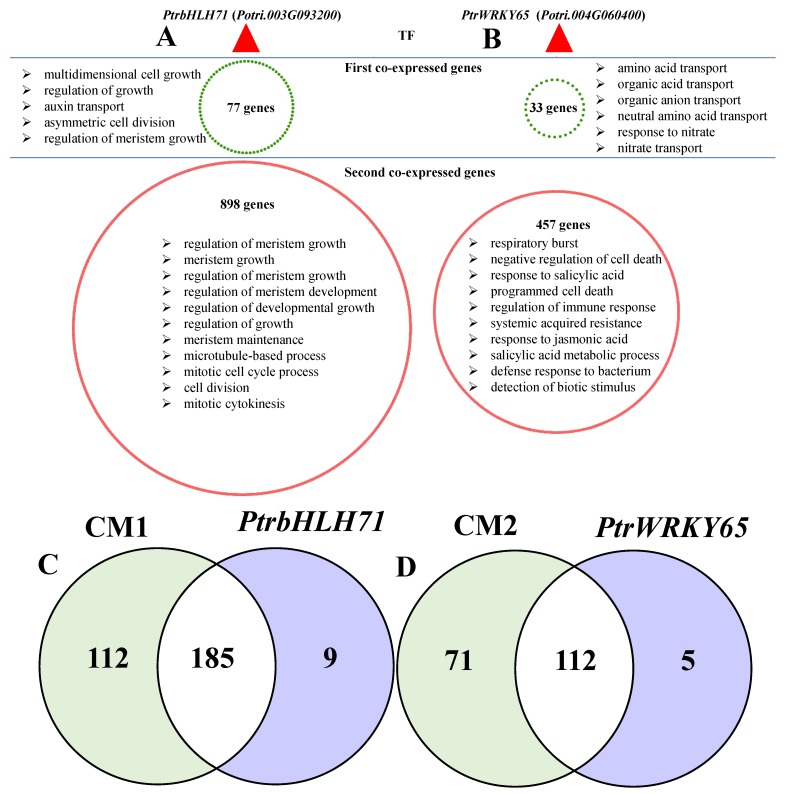
*PtrbHLH71-*centered (**A**) and (**B**) *PtrWRKY65-*centered gene regulatory networks derived from CM1 and CM2, respectively. The transcription factors and their first and second co-expressed genes are presented. The edges were hidden for the better visualization. The represented significant GO terms for the first/second co-expressed genes in the *PtbHLH71-*centered and *PtWRKY65*-centered gene regulatory networks are indicated. Venn diagram representing the overlapping significant GO terms in biological processes between CM1 and the *PtbHLH71*-centered network (**C**) and between CM2 and the *PtWRKY65*-centered network (**D**).

To further understand the regulatory mechanism underlying the coordinated regulation between plant growth and defense response in poplar, the centered regulatory network of two TFs, *Potri.003G093200* (known as *PtrbHLH71*) and *Potri.004G060400* (known as *PtrWRKY65*), were constructed. These two TFs were also classified as CRG and were derived from CM1 and CM2, respectively (Figure 7A,B). The PtrbHLH71-centered network included 976 out of 1245 genes from the co-expression network of CM1 (Figure 7A). GO term analysis of the first and second co-expressed genes confirmed the roles of this *PtrbHLH71* in regulating growth and development (Table S10). The significant GO terms of the *PtrbHLH71*-centered network shared 185 common terms with those of CM1, and almost all of the shared terms were related to growth and metabolism (Figure 7C, Tables S7 and S10). Clearly, the significantly enriched GO terms in the *PtrbHLH71*-centered network related to growth were affected by the phytohormone auxin, which was consistent with the previous conclusions. Indeed, the *PtrbHLH71* homologous gene *AtbHLH71* in *Arabidopsis* was involved in the auxin signaling pathway, which can be targeted by *ARF3* [67]. Here, genes from the *PtrbHLH71*-centered network were enriched in auxin-mediated growth processes, suggesting the possible roles of this gene in regulating growth via the auxin signaling pathway in poplar. Given that this gene was also in the CRG list, its roles in regulating both plant growth and defense response could be proposed. Similar results were also obtained from the *PtrWRKY65*-centered network, and GO term analysis revealed that similar pathways were found both in the *PtrWRKY65*-centered network and co-expression gene network of CM2 (Figure 7D, Tables S7 and S10). Interestingly, the first co-expressed genes of *PtrWRKY65* were enriched in transport, which may explain why numerous GO terms in CM2 were related to transport (Table S9). Previous studies demonstrated the roles of *AtWRKY65* in carbon starvation [68]. Furthermore, transport regulation is considered the common regulatory mechanism of plants in response to diverse nutritional deficiency responses [65,69,70]. Similarly, we identified several GO terms related to nutrition starvation in CRG (Table S7), such as “response to starvation”, “cellular response to nutrient levels”, and “cellular response to nitrogen starvation”. Thus, the defense responses regulated by *PtrWRKY65-*centered gene networks may be closely linked to changes in carbon and nitrogen metabolism. Taken together, our data clearly suggest that several TFs in the CRG might act as hub genes in the co-expressed gene network by integrating multiple hormone signaling to regulate plant defense response and growth in JA/SA-dependent manners.

**Figure 8:**
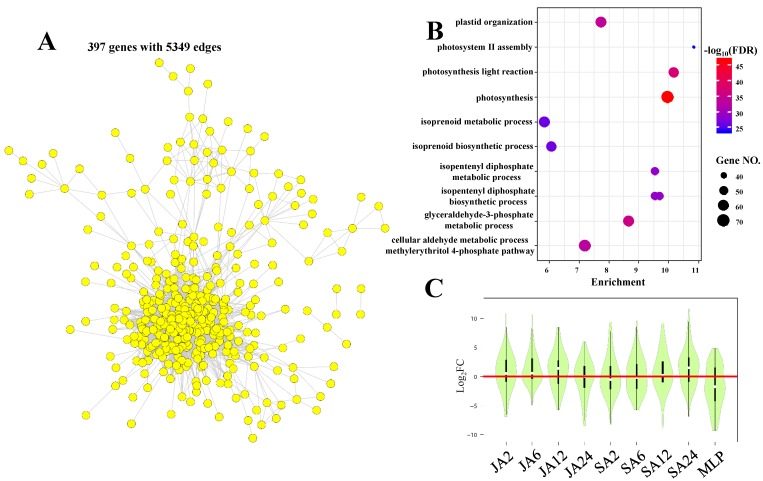
Gene co-expression networks of co-expression module four (**A**). The nodes and edges represented the genes and the significant correlations between genes, respectively. Genes without any edges were not presented in the network. The top 10 significantly enriched GO terms in biological processes in CM4 (**B**). The average expression levels of CRG from CM4 upon different treatments (**C**).

In addition to CM1 and CM2, numerous genes were also assigned to CM4 (Figure S2B, Table S8). The gene co-expression network of CM4 contained 397 genes with 5349 edges (Figure 8A). The GO term analysis of CM4 confirmed its roles in controlling photosynthesis upon fungal infection. For example, GO terms relative to the photosynthesis and biosynthesis of pigment precursors were significantly enriched in genes from CM4 (Figure 8B). Indeed, the pathways of glyceraldehyde-3-phosphate metabolic process, methylerythritol 4-phosphate, isopentenyl diphosphate biosynthetic process, and isoprenoid metabolic processes that are presented are considered as the key metabolism pathways involved in the synthesis of precursors of chlorophyll [71,72,73]. Further analyses identified 56 CRG in CM4, and their gene expression levels were globally inhibited upon MLP infection (Figure 8C). Gene ontology term analysis of these CRG from CM4 revealed consistent results, indicating that these genes generally played certain roles in controlling pigment biosynthesis (Table S11). Therefore, the significant downregulation of expression of genes that are involved in photosynthesis and the upstream pigment metabolism processes indicated reduced photosynthetic capacities upon fungal infection. These results were consistent with previous data obtained in numerous plants, including poplar [74,75]. The reduction in photosynthesis upon fungal infection is considered as an adaptive strategy for plants to switch off assimilation processes in favor of respiration processes that generate products that are required for defense [74]. Thus, the reduced photosynthetic capacities eventually resulted in constrained growth that may help plants improve their defense abilities upon pathogen attack. Again, the knowledge obtained from the co-expression analysis verified the roles of CRG in growth and defense. Taken together, our data revealed the roles of JA/SA-responsive genes in mediating photosynthesis capacities that coordinated growth and defense responses upon fungal infection.

## 4. Conclusions

**Figure 9:**
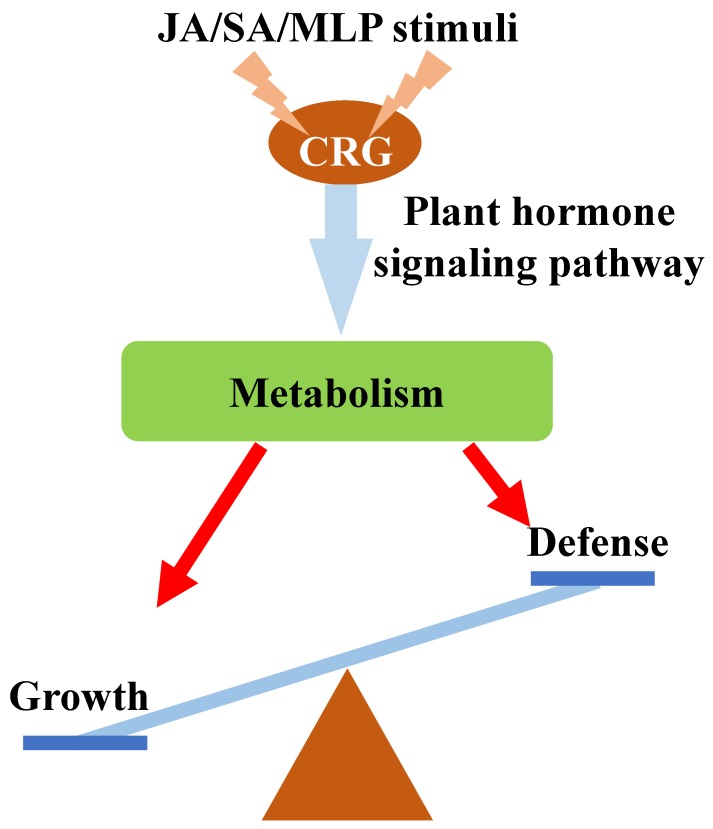
Schematic model representing the possible roles of CRG in regulating plant defense.

As summarized in Figure 9, the roles of plant hormones JA and SA during the regulation of poplar defense were characterized through integrated transcriptome analyses in this study. In total, 943 genes were identified as CRG, and these genes are generally involved in the processes of stress responses, metabolism, and growth and development. Further cluster analysis of the CRG allowed us to identify a set of core genes that are involved in the JA/SA-mediated responses to fungal defense. The different gene reprogramming profiles of the core genes upon JA/SA and MLP treatments highlighted the different roles of JA and SA on plant fungal defense regulation. The CRG modified the key pathways that are important for plant defense, such as metabolism, biotic stress, and plant signal pathways. Furthermore, co-expression analysis of JA-/SA-/MLP-responsive genes confirmed the roles of JA and SA in regulating both growth and defense responses, and their different expression profiles suggested that growth ability was reduced when poplar deployed defense responses. Several TFs of CRG in the co-expression network were proposed as hub genes in regulating these processes. Taken together, our data demonstrate the possible roles of JA/SA in regulating the balance between growth and defense responses by integrating multiple hormone signaling pathways. 

With the development of sequencing technology, increasing amounts of data will be released publicly. Therefore, it is increasingly important for researchers to make use of public data. Here, we performed a case study on how to combine experimental data with valuable information from free publicly available data. The result of this study also demonstrated the potential value of public data. However, we only collected transcriptomic data in this study. Currently, a number of different types of genomic data are available; therefore, the development of new and system-based strategies should be a major task for scientists in the future.

## Supplementary Materials

The following are available online at http://www.mdpi.com/2218-273X/9/1/12/s1, Figure S1: Correlation of qRT-PCR/RNA-Seq data for nine tested genes, Figure S2: Cluster dendrogram of 18 CMs for JA/SA/MLP-responsive genes during poplar fungal defense, Table S1: Primers used for qRT-PCR assay, Table S2: Identification of JA- and SA-responsive genes upon JA and SA treatments, Table S3: GO term and KEGG analysis of JA- and SA-responsive genes, Table S4: Identification of MLP-responsive genes, Table S5: Genes identified as the common responsive genes (CRG), Table S6: Transcription factors identified in the common responsive genes, Table S7: GO term and KEGG analysis of genes from the common responsive genes and subclusters, Table S8: Coexpression modules of JA/SA/MLP-responsive genes during poplar defense, Table S9: GO term and KEGG analysis of genes from different CMs in the coexpression network, Table S10: GO term and KEGG analysis of the *PtrbHLH71*- and *PtrWRKY65*-centered gene networks, Table S11: GO term and KEGG analysis of the CRG in CM4.
