# Supplementary material for: Integrated Transcriptome Analysis Reveals Plant Hormones Jasmonic Acid and Salicylic Acid Coordinate Growth and Defense Responses upon Fungal Infection in Poplar
Source: Biomolecules. 2019 Jan 2;9(1):12. doi: 10.3390/biom9010012 (PMC6358764; doi:10.3390/biom9010012)
Supplement: Supplementary file 1 [file biomolecules-09-00012-s001.zip › Supplemental figures.pdf]

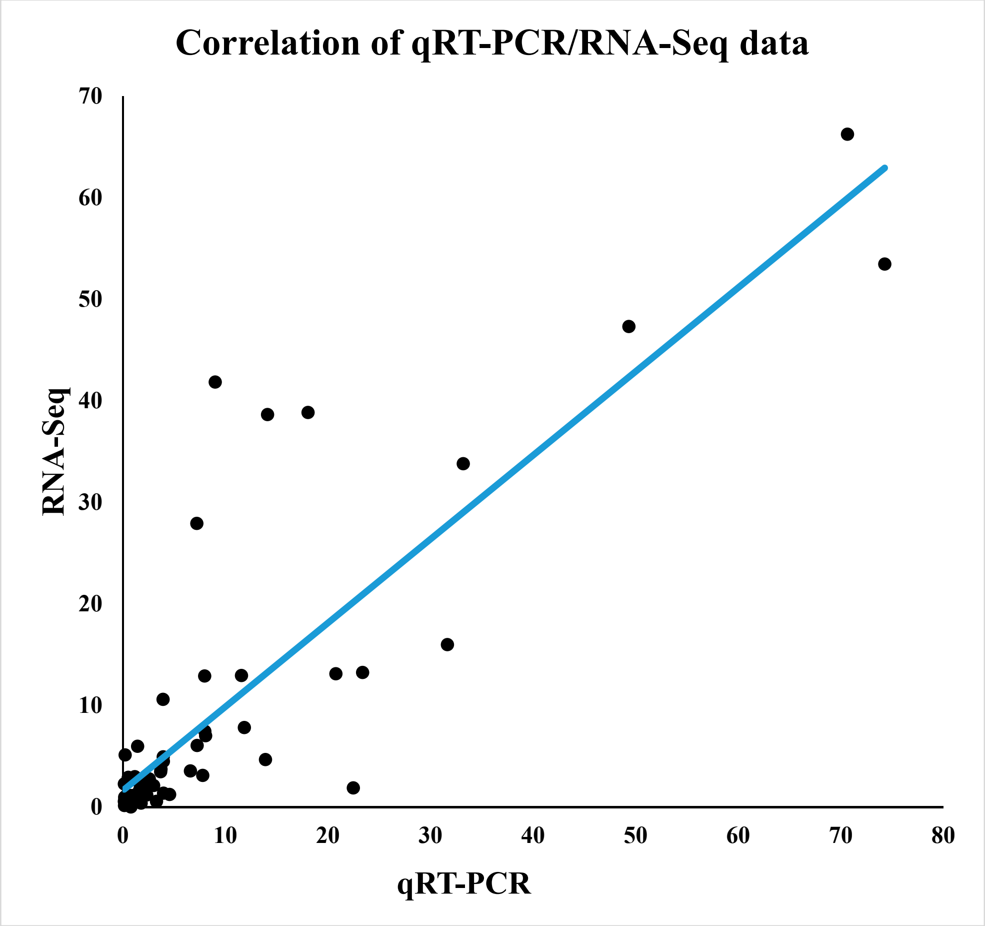

Figure S1. Correlation of qRT-PCR/RNA-Seq data for 9 tested genes.

A

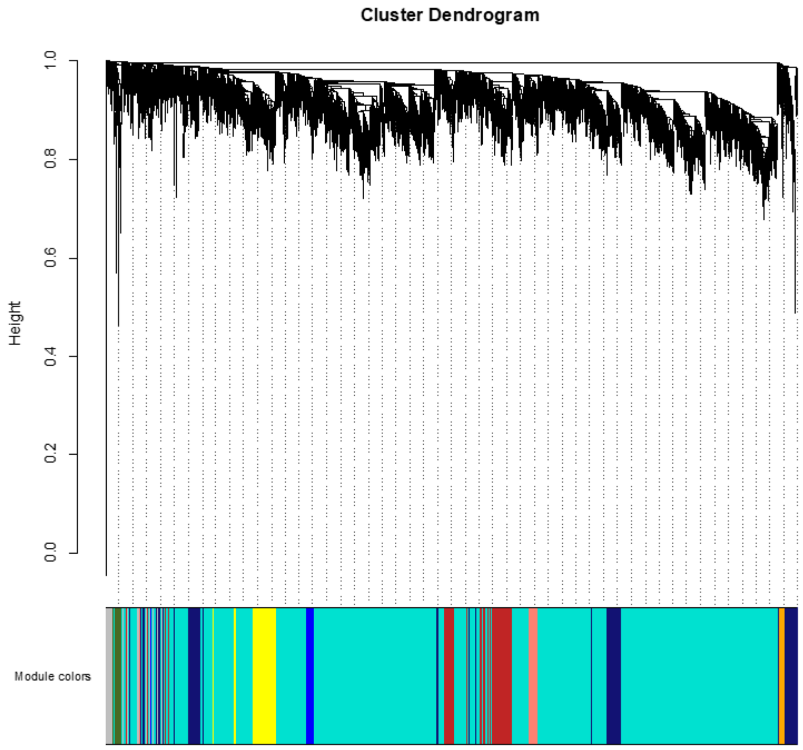

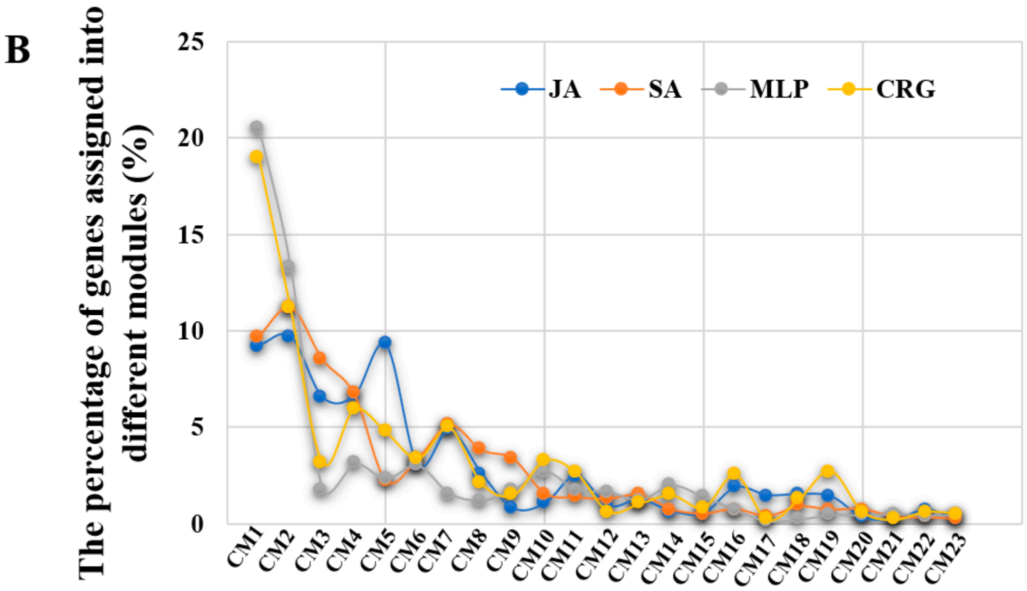

Figure S2 Cluster dendrogram of 18 CMs for JA/SA/MLP-responsive genes during poplar fungal defense. Hierarchical clustering dendrogram of the average network adjacency for the identification of co-expression modules (CMs). Genes in the same CM show the same color. The percentage of genes assigned into different CMs for JA/SA/MLP-responsive genes and common responsive genes (CRG, B).
